# Molecular Identification, Pathogenesis, and Life Cycle of *Sarcocystis cruzi* from Cattle (*Bos taurus*) in New Valley Governorate, Egypt

**DOI:** 10.1155/2023/7829290

**Published:** 2023-03-08

**Authors:** Mohammed B. M. El-Mahdi, Soheir A. Rabie, Reda M. El-S. Hassanine, Amal A. Hassan, Obaida F. Abo Elhussien, Mamdooh Ghoneum, Mohamed S. A. El-Gerbed

**Affiliations:** ^1^Molecular Genetic & Molecular Biology Lab, Zoology Department, Faculty of Science, South Valley University, Qena, Egypt; ^2^Zoology Department, Faculty of Science, South Valley University, Qena, Egypt; ^3^Zoology Department, Faculty of Science, New Valley University, El-Kharga, Egypt; ^4^Zoology Department, Faculty of Science, Damanhur University, Damanhur, Egypt; ^5^Department of Surgery, Charles Drew University of Medicine and Science, Los Angeles, CA 90059, USA; ^6^Department of Surgery, University of California Los Angeles, Los Angeles, CA 90095, USA

## Abstract

*Sarcocystis cruzi* was identified by molecular methods from an intermediate host, cattle (*Bos taurus*), in El-Kharga, New Valley Governorate, Egypt, and its life cycle and pathogenicity were studied in the final host, dogs (*Canis familiaris*). 600 slaughtered cattle aged 6–8 years (480/120 males/females) were included. In addition, three laboratory-bred, coccidian-free puppies aged 2–3 months were fed infected bovine muscles to locate the definitive host and analyze sporogony. 18S rRNA-specific gene primers were used for DNA amplification from esophageal muscles. These polymerase chain reaction (PCR) amplicons were subjected to restriction fragment length polymorphism (RFLP) and molecular sequence analysis. Infection was detected in 78.8% (473/600; 95% CI, 75.56–82.11%). Histopathological examination of esophageal muscles showed oval- to spherical-shaped cysts, 96.7 *μ*m wide by 326.9 *μ*m long; cysts in cardiac muscles were ovoid and smaller. Infected puppies began shedding sporocysts in feces 7 days post-inoculation and showed distorted organ architecture, severe cellular damage, and inflammatory lesions in liver, kidney, esophagus, and stomach. Three oocysts with different shapes and sizes were identified. Partial 18S rRNA gene sequences of isolated New Valley sarcocysts were identical to *S. cruzi* isolated from different areas, verifying their genetic relatedness. Our analysis suggests that *S. cruzi* is the most prevalent in slaughtered cattle in New Valley Governorate, Egypt.

## 1. Introduction

Apicomplexan parasites of the genus *Sarcocystis* have an obligate two-host life cycle and comprise about 200 species that infect different cold- and warm-blooded hosts, including humans [1]. With their two-host life cycle, they have prey and predator hosts serving as intermediate and definitive hosts, and omnivores serving both hosts [2]. The life cycle of *Sarcocystis* has been extensively studied [3]. *Sarcocystis* species have many domestic herbivorous animals that act as prey or intermediate hosts. These include cattle, ox, buffalo, horses, sheep, goats, and pigs [4, 5]. Cattle have been shown to serve as the intermediate host for several *Sarcocystis* species, such as *Sarcocystis cruzi*, *Sarcocystis heydorni*, *Sarcocystis hirsuta*, *Sarcocystis hominis*, and *Sarcocystis rommeli* (formerly known as *S. rommeli*-like), with different definitive hosts, including canids, felids, and humans [3]. In cattle, three species are recognized with known endogenous stages: *S. cruzi* (with canine definitive host), *S. hirsuta* (feline definitive host), and *S. hominis* (primate definitive host). Single carnivores, such as cats and dogs, have also been recognized as capable definitive hosts for a variety of parasites [6]. Earlier studies showed that *S. hominis* infected cattle may cause symptoms in humans, including symptomatic gastroenteritis, which presents as nausea, abdominal pain, and watery diarrhea [3]. Therefore, the infection is potentially zoonotic, that is, the animal parasites can infect humans. Consuming raw or undercooked beef containing mature cysts can be considered as potential infecting agents to humans, whose infections are distributed worldwide [7–9].

Distinguishing between species has often been performed through morphological characterization of *Sarcocystis* with light or transmission electron microscopy (TEM) [2, 10, 11]. However, these methods of analysis are not completely accurate because the appearance of *Sarcocystis* may change in accordance with the location and the developmental stage of cysts. Further studies proposed differentiating between *Sarcocystis* species based on their walls, which vary significantly in their thickness [2, 12]. In addition to accuracy, the cost and time consumption of TEM can be a limiting factor in its frequent use. Molecular approaches have therefore been suggested for identification of morphological species, since they allow for quick and accurate identification of *Sarcocystis* species in cattle [13, 14]. DNA sequencing, cox1, and ITS1 gene sequences are valuable means for identifying and characterizing *Sarcocystis* species, particularly when regions of the 18S rRNA gene can be utilized and analyzed, even from the same genus [6]. The current work aims to describe the molecular identification, pathology, and life cycle of *S. cruzi* detected from slaughtered cattle in El-Kharga, New Valley Governorate, through TEM, 18S rRNA polymerase chain reaction (PCR) analysis, sequencing, and molecular phylogeny. Additionally, pathological changes in some organs of the definitive host were investigated.

## 2. Materials and Methods

### 2.1. Ethics Approval

All animal experiments followed the institutional ethical guidelines for the care and use of animals in research to the letter. Approval for animal studies was obtained from the Faculty of Science, South Valley University, Qena, Egypt (Approval number: 22/18 10.2021).

### 2.2. Clinical Signs and Detection of *S. cruzi* in Cattle

Clinical signs for each cattle (*Bos taurus*) were observed by a veterinary physician. Fresh samples were also examined under a microscope for the existence of *Sarcocystis* cysts [15].

### 2.3. Sample Collection and Histological Processing

Between February 2018 and February 2021, a fresh part of esophageal and cardiac muscle samples (about 50 g) from each of the 600 naturally infected cattle (*B. taurus*) of both sexes and 6–8 years of age were obtained from a slaughterhouse located in El-Kharga, New Valley Governorate, Egypt. In addition, for histopathological studies, infected dogs were humanely euthanized using pentobarbital administered intravenously. The fresh chosen organs (liver, kidney, esophagus, and stomach) were collected. All samples were washed with normal saline solution and fixed in 10% neutral buffered formalin for 24 hours. Specimens were dehydrated using ascending ethyl alcohol concentrations, double cleared in xylene, and embedded in paraffin. Serial cuts were sectioned at 5 *μ*m using a rotary microtome and stained with hematoxylin and eosin (H&E) for histological and histopathological examination [16]. The remainder of the cattle samples were stored at −20°C for PCR.

### 2.4. Transmission Electron Microscope

Pieces from positive muscles confirmed to have infection by light microscopic examination were fixed with 2.5% glutaraldehyde solution in 0.1 M phosphate buffer (pH 7.4) for 4 hours. After the buffer wash, specimens were post-fixed with 1% osmium tetroxide at 4°C for 4 hours. Afterward, the specimens were dehydrated in a graded ethyl alcohol series with two changes of propylene oxide and embedded in an Epon mixture. Before being examined with a JEOL JEM-1010 TEM (Japan), the ultrathin slices were stained with uranyl acetate and lead citrate.

### 2.5. Experimental Infection

Three young puppies (*Canis familiaris*) aged 2–3 months were laboratory-bred and coccidian-free in order to detect sporogony. Puppies used in the experimental infection were usually fed boiled milk and bread and had never been fed meat before infection. Two were fed small segments of highly infected muscles of cattle and one used as a control. Fecal samples from these dogs were examined daily using light microscope for shedding of any coccidian oocysts for a period of 8 weeks post-infection.

### 2.6. Molecular Studies

*Genomic DNA Extraction.* Esophageal muscle tissues were removed from slaughtered cattle (*B. taurus*) and frozen to be used for molecular analysis. Genomic DNA was obtained from frozen cyst-positive samples to be identified with light microscopy. DNA was extracted using DNeasy Blood & Tissue Kits (Qiagen, Hilden, Germany) following the manufacturer's instructions. For PCR analysis, the DNA samples were kept at −20°C.

*Amplification, RFLP, and Sequencing of Partial 18S rRNA Gene.* To amplify a partial region of the 18S rRNA, primers Sar F 5′; GCA CTT GAT GAA TTC TGG CA 3′; and Sar R 5′;CAC CAC CCA TAG AAT CAA G′; 3 [17] were used. These are specific for detecting the cattle *Sarcocystis* species. The PCR reaction was performed in BOECO (thermal cycler TC-TE) in a total volume of 25 *μ*l comprising 3 *μ*l DNA sample, 20 pmol of each primer (2 *μ*l), 12.5 *μ*l of PCR Master Mix, and 7.5 *μ*l distilled water. The cycling conditions included 94°C for 5 minutes followed by 40 cycles of 94°C for 2 minutes, annealing at 55°C for 1 minute, and extension at 72°C for 90 seconds, followed by a 5-minute final extension at 72°C. PCR bands were separated on 1% agarose/ethidium bromide, visualized under UV light, and photographed using a digital camera. Following DNA amplification, digestion of PCR products was performed according to the manufacturer's instructions by restriction fragment length polymorphism (RFLP) method utilizing *Moraxella bovis* (Mbo1) and *Haemophilus influenza* Rf (Hinf) endonuclease enzymes. 5 *μ*l of the PCR reaction was mixed with 10 units of enzyme, 2 *μ*l of 10× buffer, and 18 *μ*l distilled water. The mixture was centrifuged for 30 seconds and then incubated for 24 hours at 37°C [18].

PCR products from chosen samples were purified using QIAamp PCR Purification Kit (QIAGEN) according to the manufacturer's instructions and then were sequenced in both directions (GATC Biotech DNA Sequencing, Germany) with the same primers used for amplification. Obtained DNA sequences were compared with available cattle *Sarcocystis* spp. DNA sequences for verification and homology. For phylogenetic analysis, obtained DNA sequences and other selected cattle *Sarcocystis* spp. (16 sequences) were analyzed using MegaX software [19]. Tree reconstruction was carried out using the maximum likelihood [20] under distance measurement of Tamura 3-parameter with gamma distributed rate (T92 + G + I). The statistical support for branches on the muscular layer (ML) tree was determined by 1000 bootstrap replicates [21]. The *Sarcocystis anasi* (EU553477, *Anas platyrhynchos*, 1792 bp, Lithuania, leg muscles) and *Sarcocystis falcatula* (MH626537, Rainbow Lorikeets, 1646 bp, USA, lung) were used as outgroups.

## 3. Results

### 3.1. Clinical Signs of Infected Cattle

Signs in cattle acutely infected with *S. cruzi* include fever, anorexia, decreased milk yield, diarrhea, loss of tail hair, muscle spasms, hyperexcitability, general weakness, and prostration.

### 3.2. Histopathological Examination of *S. cruzi*-Infected Cattles' Esophageal and Cardiac Muscles

**Figure 1:**
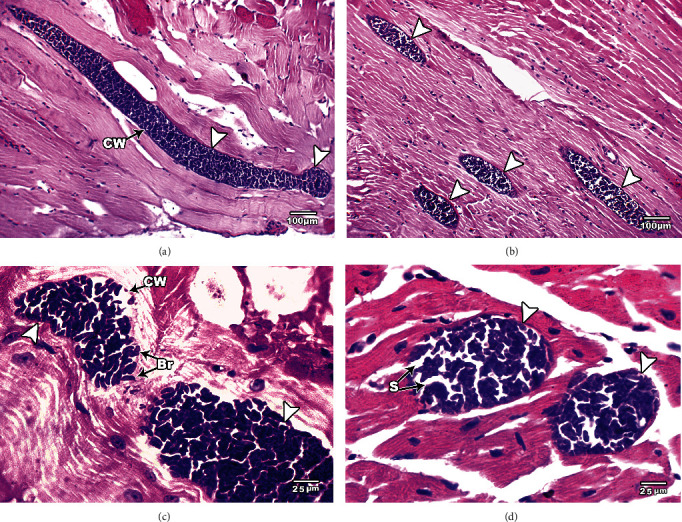
Light micrographs of *S. cruzi* in muscle tissues of cattle, hematoxylin and eosin-stained images. Elongated sarcocysts are seen embedded in the esophagus (a–c) and cardiac (d) muscles. Arrows refer to the sarcocyst. Note the cyst wall (CW) underlined by a layer of ground substance extended into the interior of the cyst as septa dividing it into compartments enclosing bradyzoites (Br). Original magnification: 100× (a and b) and 400× (c and d).

Figure 1 shows the histopathological examination of *S. cruzi*-infected cattle's esophageal and cardiac muscles. *Sarcocystis* could not be detected macroscopically during the postmortem examination of the slaughtered cattle. Histopathological examination of esophageal and cardiac muscle samples from the slaughtered cattle revealed sarcocysts in 78.8% (473/600; 95% CI, 75.56–82.11%) and 60.17% (361/600; 95% CI, 56.24–64.10%), respectively. Light microscopy shows the cysts to be oval to spherically shaped. The sarcocysts appeared in the esophagus with fusiform or oval shapes and sizes of 96.7 *μ*m wide by 326.9 *μ*m long (Figures 1(a), 1(b), and 1(c)), while the sarcocysts in the cardiac muscles were ovoid and smaller in size, with an average of 48.8 *μ*m wide by 158.1 *μ*m long (Figure 1(d)). Both sarcocysts' walls were thin (<1 *μ*m thickness). Some cysts were septate, and the cyst cavity was divided into many chamberlike compartments separated from each other by septa derived from the ground substance located under the primary cyst wall. The interior compartments were packed with bradyzoites (Figures 1(a), 1(b), 1(c), and 1(d)).

### 3.3. *S. cruzi* Ultrastructural Study

**Figure 2:**
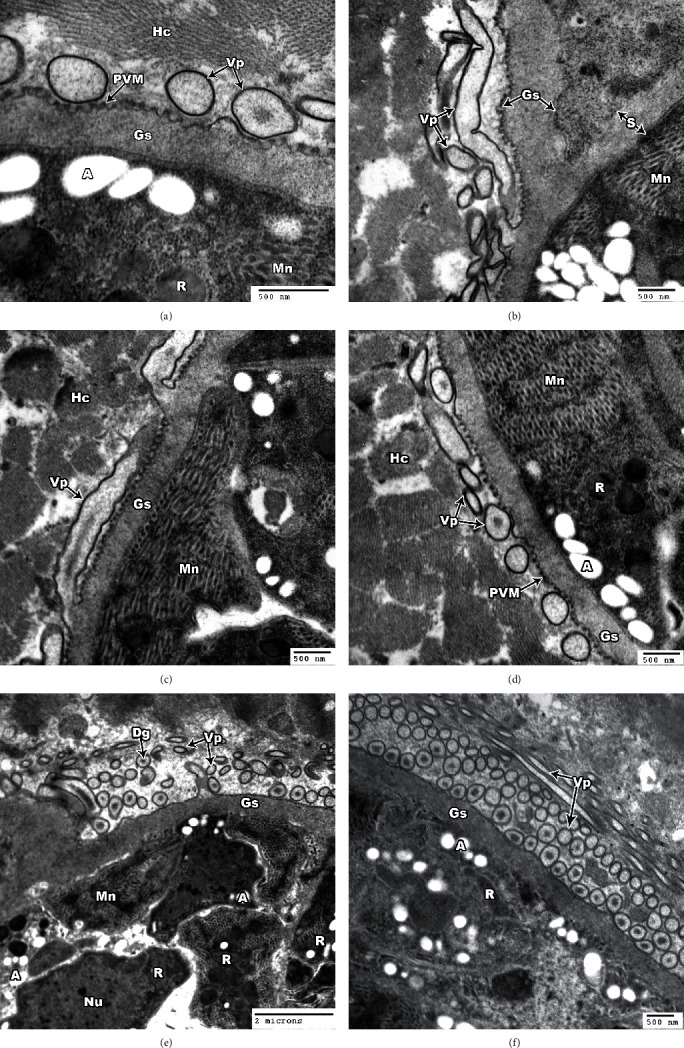
TEM of *S. cruzi* wall. (a) Parasitophorous vacuolar membrane (PVM) and villar protrusions (Vp). (b) Villar protrusions (Vp) and ground substance (Gs). (c) Cyst wall protrusions (Vp) with no microfilaments. (d) Cyst wall protrusions (Vp) adjacent to the sarcoplasm of host cell (Hc). Note magnified protrusion. (e) Cyst wall with hair-like protrusions (Vp); the protrusion contains coarse or fine electron dense granule (Dg). (f) Villar protrusions cut in different directions.

**Figure 3:**
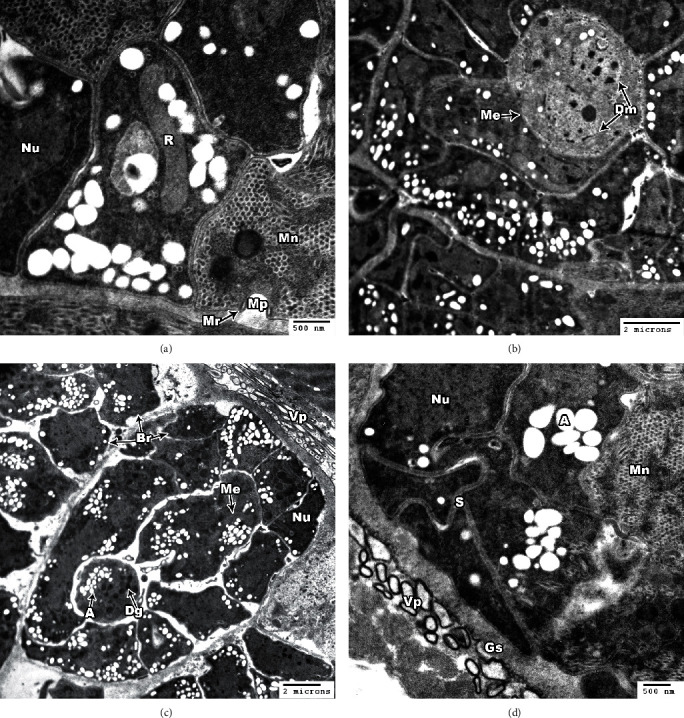
TEM of *S. cruzi*. (a) Section through the micropore (Mp); the pore is surrounded by a micropore ring (Mr). Note the clear micronemes (Mn), nucleus (Nu), and rhoptry (R). (b) The architecture of a dividing metrocyte (Me) with two daughter merozoites (Dm) in the inside of a *Sarcocystis cruzi* cyst. (c) A high concentration of bradyzoites is enclosed by a thin cyst wall and septa. Note the bradyzoites containing several dense granules (Dg), in addition to several amylopectin granules (A), nucleus (Nu), and mitochondrion (Mc). (d) Cross section of bradyzoite showing nucleus (Nu), amylopectin granules (A), micronemes (Mn), septa (S), and ground substance (Gs).

Ultrastructural analysis of *S. cruzi* is illustrated in Figures 2 and 3. The cyst wall is thin (<1 *μ*m in width) and consists of a parasitophorous vacuolar membrane (PVM), which is characterized by irregular undulation (irregularly spaced pits interrupted at irregular intervals) (Figures 2(a) and 2(b)). The membrane is provided with hair-like villar protrusions (Vp) folded over the surface of the cyst. These protrusions are rod, round, oval, or irregular in shape according to their sections, and they are approximately parallel to the cyst surface (Figures 2(b), 2(c), 2(d), 2(e), and 2(f)). Each protrusion contains coarse or fine electron dense granule (Dg) with no fibrillar structure (Figure 2(e)). The protrusions arise irregularly from the base, contain a fine granular substance, lack internal microfilaments (Figures 2(c) and 2(e)), and measure approximately 0.69–3.78 *μ*m in length and 0.13–0.45 *μ*m in width. The ground substance (Gs) measures approximately 0.375–0.762 *μ*m in length. Bradyzoites (Br) were found in the inner side of the ground substance and measure 3.80–9.73 *μ*m × 3.70–6.87 *μ*m (Figure 3(c)). The bradyzoites contain rhoptries (R) and a large number of micronemes (Mn), nuclei (Nu), and abundant amylopectin granules (A) (Figures 3(a), 3(c), and 3(d)). In some bradyzoites, the micropore (Mp) appears surrounded by a micropore ring (Mr) (Figure 3(a)). Some metrocytes (Me) have two daughter merozoites (Dm) (Figure 3(b)).

### 3.4. *S. cruzi* Life Cycle

We noted that the experimentally infected puppies began to shed sporocysts in the feces 7 days post-inoculation with diarrhea, and this persisted until day 15–17 days post-inoculation at the same rate. The sporocysts remained excreted but in low number until 30 days post-inoculation. The sporocysts then multiplied in quantity and continued to pass until the experiment end (60 days post-inoculation).

### 3.5. *S. cruzi* Oocyst's Shapes and Sizes

**Figure 4:**
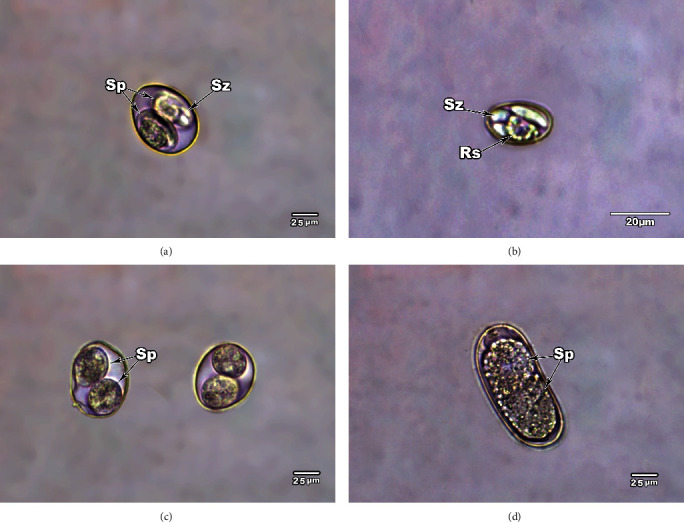
Unstained mature globular oocysts of *Sarcocystis* from feces of the infected dogs. (a) Oocysts with two elongated sporocysts (Sp), which lie transversely in their position inside the oocyst. Note the sporozoites (Sz) inside the sporocyst. (b) Elongated ellipsoidal sporocyst, with each sporocyst contain four sporozoites (Sz) and one large internal residual body (Rs). (c) Oocyst containing two rounded sporocysts, which lie longitudinally at their position inside the oocyst. (d) Oocyst with two incompletely divided sporocysts, which were composed of numerous small granules. Original magnification: 400×.

Oocysts enter the intestinal lumen and exit the body through the feces of infected dogs, with each oocyst containing two sporocysts. Three types of mature oocysts could be identified; they are shown in Figures 4(a), 4(b), 4(c), and 4(d). The first type of oocyst is globular, with two oval, elongated sporocysts lying transversely in their position inside the oocyst. The oocyst wall is thick, and the oocyst measures 23.71–27.48 *μ*m in length (mean 25.60 *μ*m) and 20.24–23.62 *μ*m in width (mean 21.93 *μ*m) as in Figure 4(a). Inside the oocyst, ellipsoidal sporocysts appear, each with four sporozoites (Figure 4(b)).

The second type is ellipsoidal in shape and contains two rounded sporocysts lying longitudinally in their position inside the oocyst, and the oocyst measures 24.75–26.73 *μ*m in length (mean 25.74 *μ*m) and 20.60–21.38 *μ*m in width (mean 20.99 *μ*m) as in Figure 4(c). The third type is oval in shape and contains two unseparated elongated sporocysts lying longitudinally in their position inside the oocyst, and the oocyst measures 64.42–65.35 *μ*m in length (mean 64.88 *μ*m) and 27.62–29.06 *μ*m in width (mean 28.34 *μ*m) as in Figure 4(d).

### 3.6. Histopathological Examination

Histopathological examination using light microscopy was carried out for different organs of infected dogs. These included liver, kidney, esophagus, and stomach.

#### 3.6.1. Infected Dogs' Liver

**Figure 5:**
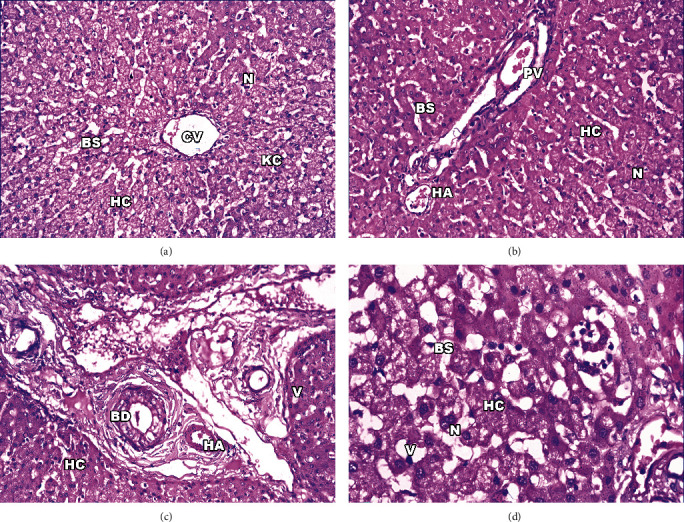
Light micrograph of infected dogs' liver sections. (a) Hepatocytes (Hc) with variable size and shape nuclei (N). Note the dilated central vein (Cv), blood sinusoid (Bs), and activated Kupffer cells (Kc). (b) Hepatocytes (Hc) with nuclei (N), hepatic portal vein (Pv), and blood sinusoid (Bs). (c) Portal area with dilated and congested portal vein (Pv) and numerous branches of bile ducts (Bd). (d) Disorganized liver hepatocytes (Hc) with cytoplasmic vacuolization (V). Original magnification: 400×.

Examination of liver sections obtained from infected dogs revealed distortion of the liver architecture, as illustrated in Figure 5. Hematoxylin and eosin-stained liver sections revealed hepatocellular injury represented by the loss of the normal architecture of the liver. This was indicated by many hepatocytic and sinusoidal alterations. Others lost their regular shape and size, while cellular pleomorphism was also observed. The hepatocyte nuclei displayed pale-violet stain and had a pleomorphic appearance. The majority of the core veins showed a noticeable dilatation. Cellular necrosis was visible in the hepatocytes after further inspection. Portal veins that were dilated and crowded as well as numerous bile duct branches were present in the portal locations (Figures 5(b) and 5(c)). When the central vein and sinusoids were observed, they had irregularity in the border architecture and frequently appeared ripped. Additionally, cytoplasmic degradation, necrotic foci, activation of the Kupffer cells, bleeding, and infiltration of inflammatory cells were seen (Figures 5(b) and (d)).

#### 3.6.2. Infected Dogs' Kidney

**Figure 6:**
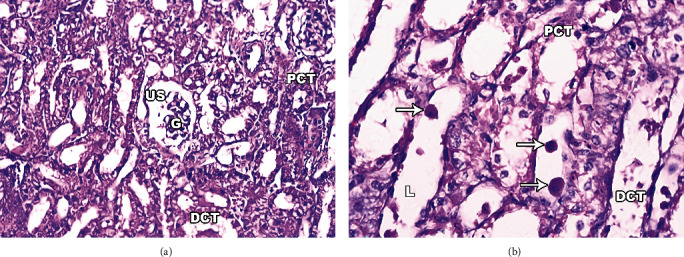
Light micrographs of infected dog's kidney sections stained with H&E. (a) Section of renal cortex showing highly deteriorated renal corpuscle with degenerated glomerulus (G), dilated urinary space (US), and proximal (PCT) and distal (DCT) convoluted tubules. (b) Kidney tissue with cellular abnormalities and leukocyte infiltration. Note the tubular necrosis, epithelial lining degeneration, necrotic foci, distorted proximal (PCT) and distal (DCT) convoluted tubules with dilated lumen (L), and segregated nuclei with red blood cells (RBCS) (arrows) in its lumen. Original magnification: 400×.

The architecture of the nephron, the body's fundamental structural unit, was clearly altered and deteriorated upon examination of kidney sections from infected dogs stained with H&E. The pathologic changes showed signs of cellular damage (loss of normal staining) and several cellular abnormalities, including extensive bleeding and cellular infiltration in the interstitial tissue of the renal cortex, which had a necrotic appearance. Additionally, the light micrographs showed that inflammatory cells had infiltrated the glomeruli, giving them the appearance of being hypertrophied. The Bowman's space's (urinary space's) most noticeable aberration was that it appeared greatly enlarged. The renal tubules, on the other hand, showed signs of damage and deterioration. The proximal convoluted tubule (PCT) (brush border) surface luminal specialisation appeared distorted and fractured (Figures 6(a) and 6(b)). The lumens in PCT and distal convoluted tubule (DCT) are similar.

#### 3.6.3. Infected Dog's Esophagus

**Figure 7:**
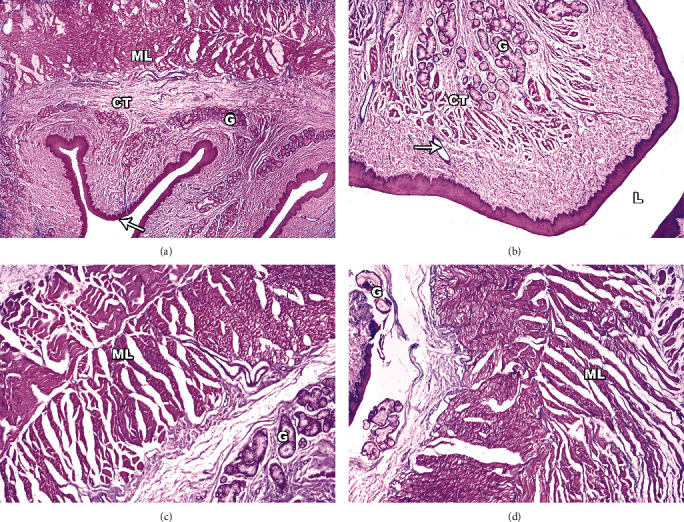
Light micrographs of infected dogs' esophagus sections stained with H&E. (a) Full-thickness section of esophagus showing four layers: mucosa (thin arrow), submucosa, muscular layer (ML), adventitia and submucosal glands (G), and ducts and keratinized stratified squamous epithelium (small arrow). (b) Marked degenerative changes of muscle fibres with dispersing edema dispersing muscle fibres and esophageal glands (G) located in submucosa form ducts (arrow) separated by connective tissue (CT). (c) Muscular layer (ML), esophageal mucous glands (G), and connective tissue separating between glands. (d) Hypertrophied muscle fibres (ML) and damage of myofibrils. Note the increased space between the myofibrils. Original magnification: 400×.

Sections of dogs' esophaguses that had *S. cruzi* infections were examined under a light microscope and revealed several changes (Figure 7). The mucosal epithelial lining of the esophagus had substantial hyperplastic growth, according to histopathological analysis. An esophageal lesion called tunica musculosa had coagulative necrosis of the muscle fibres, inflammatory cell infiltration, and pronounced spreading edema between the muscle fibres. Tissue dilated submucosal glands and gland ducts, bordered with cuboidal or squamous epithelium, with a significant surrounding inflammatory infiltration made up of lymphocytes, plasma cells, and eosinophils, are the earliest light microscopic indications of cellular injury.

#### 3.6.4. Infected Dog's Stomach

**Figure 8:**
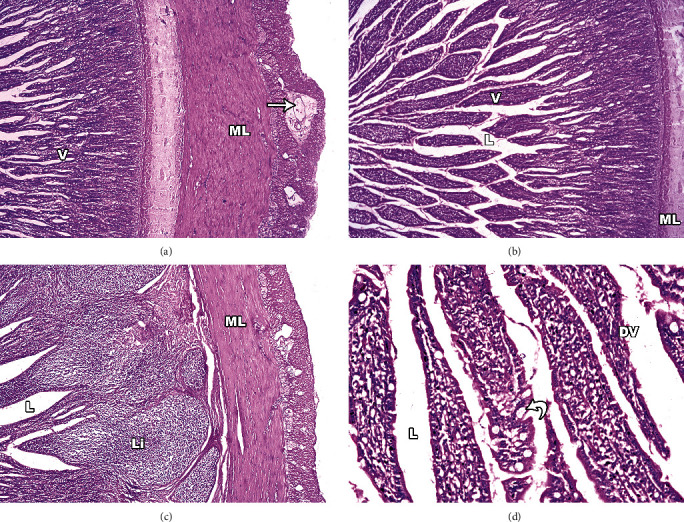
Light micrographs of infected dogs' stomach sections stained with H&E. (a) Full-thickness section of stomach showing sloughing of the simple columnar epithelium (E), absence of the normal villus (V) architecture, with damaged muscular layer (ML) and degenerated muscle fibres with marked edema (arrow). (b) Some villi show distorted architecture with dilatation of their middle part. (c) Marked leucocytic infiltration (Li) and edema widen the submucosa. (d) Dissolved villi tips with damaged villi (DV); some oxyntic cells are shrunken with pyknotic nucleus, while others are vacuolated. There is also widening of gastric glands (black arrow).

Edema and leucocyte infiltration of the submucosal layer were seen when *S. cruzi* infection was examined under a microscope in the four areas of the stomach's interior surface in infected dogs (Figure 8). H&E-stained stomach sections showed exfoliated cells in the lumen of the stomach along with loss of the mucous layer and sloughing of the surface epithelium. The height of the epithelial columnar cells had diminished, and the nuclei had lost their typical polarity, resulting in pyknotic nucleoli that were irregular. In the stomach, there were ulcers and mucositis. Lamina propria also revealed a significant number of inflammatory cells, primarily lympho-mononuclear. The architecture of the glands in the glandular stomach was deformed, and the glands' lumen was enlarged (Figure 8(a)), with some oxyntic cells shrinking and developing pyknotic nuclei.

### 3.7. Molecular Studies

#### 3.7.1. Amplification, Sequencing, and RFLP Analysis of Partial 18S rRNA Gene

**Figure 9:**
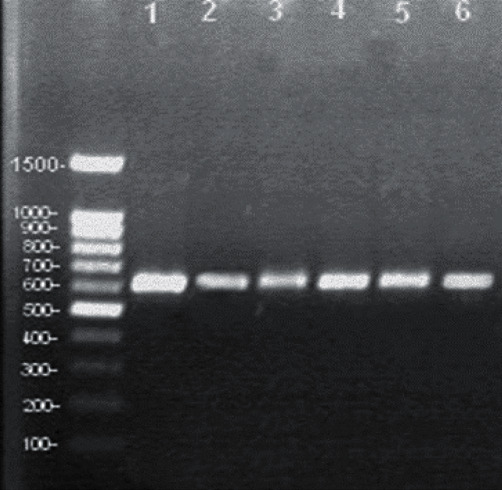
Amplification of 18S ribosomal gene of *Sarcocystis* sp. from selected random cattle samples, which demonstrate a band of 620 bp with similar length in all investigated six samples used that belonged to *S. cruzi*. The 100 bp DNA ladder Ready-to-Use (RTU) is shown.

**Figure 10:**
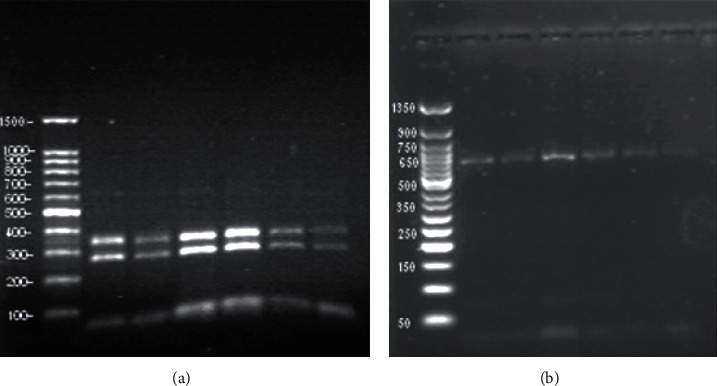
The RFLP restriction digest analysis by Mbo1 endonuclease enzyme on the amplified 18S targeted region from *S. cruzi*. (a) The Mbo1 enzyme produced two fractionated regions of approximately 275 and 350 base pair in length. The 100 bp DNA ladder Ready-to-Use (RTU) is shown. (b) The Hinf enzyme has no cutting effects. The BERUS 50 bp DNA ladder is shown.

The set of primers used (SarF and SarR) were efficiently amplified for a partial region from the 18S rDNA gene of the 620 base pair (Figure 9) from six different DNA samples of *Sarcocystis* sp., in accordance with the predicted size using these primers. The RFLP analysis utilizing *M. bovis* (Mbo1) endonuclease enzyme produced two fragments of approximately 275 and 350 base pairs (Figure 10, left), while in the case of using the *Hinf* enzyme, no cutting effects were observed (Figure 10, right).

#### 3.7.2. Sequence and Phylogenetic Analysis of *S. cruzi* Identified

After excluding the primers' sequences, we obtained a partial DNA sequence of 18S rRNA with a length of 572 nucleotides, in accordance with the expected amplified size. The obtained DNA sequence was compared and verified as being derived from *Sarcocystis* spp. Analysis of the 18S rRNA gene from *Sarcocystis* cysts from cattle (New Valley Governorate) revealed homology and similar identity (100%) with the corresponding sequences of *S. cruzi* (LC171828.1, KJ917935.1, KJ917906.1, JX679467.1, AB682779.1, and AF176933.1). The nucleotide sequence from this study has been deposited in GeneBank with accession number OL305830.

**Figure 11:**
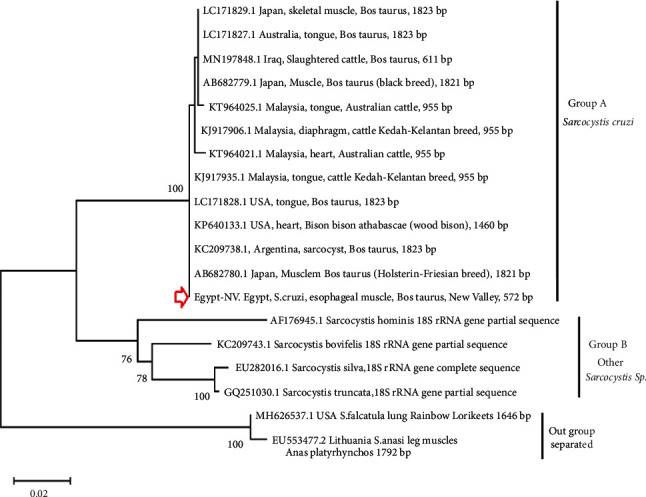
Phylogenetic analysis of *S. cruzi* isolated in the present study along with different *Sarcocystis* spp. recorded in cattle from other countries. The program MegaX V.10.2.6 was used to construct the tree using maximum likelihood method based on 18S rRNA gene sequences and under distant measurement of T92 + G + I. The Egypt-NV obtained nucleotide sequence has been deposited in the GenBank with accession number: OL305830.

The phylogenetic tree (Figure 11) of the 18S rRNA sequence revealed that the identified sarcocysts under study are closely clustered and branched with the group of *S. cruzi* from other countries (Group A). This reflects the closest identities between the *Sarcocystis* spp. under study with the previously identified *S. cruzi* from other countries, similar to those revealed in the genetic distance estimation of Table 1. Other included *Sarcocystis* spp. assembled together (Group B). Additionally, individuals included as an outgroup were clearly separated.

## 4. Discussion

*Sarcocystis* is an intracellular protozoan parasite that may cause fatal disease for its host [2]. There are six main species of *Sarcocystis* in cattle (*S. cruzi*, *S. hirsuta, S. hominis, S. heydorni*, *S. rommeli*, and *S. hjorti*), with *S. cruzi* being the most common and important. *Sarcocystis cruzi* infection in cattle is usually subclinical, although its acute and chronic clinical diseases have been described in natural and experimental cases [22]. The present study describes for the first time the complete development and long-term maintenance of *S. cruzi* of both cattle and dog genotypes in New Valley Governorate, Egypt. We describe the major symptoms of cattle infected with *S. cruzi*, including severe illness, fever, anorexia, diarrhea, muscle spasms, and loss of tail hair. These symptoms are in accordance with Dubey et al. [22]. In addition, we did not find macroscopic sarcocysts in any of the cartel samples examined, in agreement with Dubey [23].

In the present study, both morphological and molecular examinations were utilized to identify *Sarcocystis* infection in cattle. Light microscope examinations revealed that 78.8% of esophageal and 60.2% of cardiac muscle samples were positive for sarcocysts from 600 samples. The high infection rate of *Sarcocystis* spp. among slaughtered cattle was consistent with past reports in Egypt [24, 25, 26] and in agreement with the morphological examinations of others [11, 27–29]. Sarcocysts described by light microscopy and TEM vary in morphology according to species and age [30], and *S. cruzi* also shows different shapes and lengths [10, 14, 31–34]. Further studies have demonstrated that the architecture of the main cyst wall is an essential criterion in determining the ultrastructural characteristics of cysts of the *Sarcocystis* species [35]. In the present study, *Sarcocystis* sp. cysts were revealed to have a thin wall provided with hair-like villar protrusion parallel to the cyst surface. These villar protrusions had electron dense granules and no fibrillar structure. The sarcocysts appeared in the esophagus with sizes 326.9 *μ*m long by 96.7 *μ*m wide and in cardiac muscles with smaller sizes 158.1 *μ*m long by 48.8 *μ*m (bradyzoites measure 3.80–9.73 *μ*m × 3.70–6.87 *μ*m), indicating that the present description of *Sarcocystis* sp. was similar in size when compared with the previous studies on *S. cruzi*.

The prevalence of *S. cruzi* infection has been reported in different countries, and cattle are known to serve as the intermediate host. The infection rate as determined by digestion and microscopic tests has shown a wide range of 44–100% in different countries such as New Zealand [36], Brazil [32], Iran [37], and India [38]. In the present study, the infection rate of *S. cruzi* was examined and recorded as a high infection rate (78.8%) in the esophagus. These results are in agreement with other Egyptian studies showing high prevalence (84–94%) of *Sarcocystis* infection in cattle for different cities in the south of Egypt such as Qena, Assiut, and Sohag [24, 39, 40]. Collier et al. [41] reported that one reason for the high prevalence of *Sarcocystis* in cattle is the assortment of a large number of hosts that shed countless resistant sporocysts; life cycles in cattle have been established in canines (*S. cruzi*), felines (*S. hirsuta*), humans (*S. hominis*), and others [25].

Several factors can influence the detection of *Sarcocystis* species as well as the risk of infection. During the examination of different tissues, including the heart, skeletal muscle, esophagus, and diaphragm, *Sarcocystis* was found only in the esophageal and cardiac muscles. Therefore, the type of tissue can considerably influence the detection of different *Sarcocystis* spp. In addition, the grazing farming system has a positive effect on the risk of *S. cruzi* infection [42]. In the present study, we recorded six species of *Sarcocystis* (*S. cruzi*, *S. hirsuta*, *S. rommeli*, *S. hjorti*, *S. bovifelis*, and *S. fusiformis*). Among these, *S. cruzi* was found to have the highest prevalence, and this species was therefore identified and investigated in detail.

In experimental infections of *S. cruzi*, the pattern of discharge of sporocysts in dog feces has been reported [43]. Heart muscles from cattle fed to pups and kittens resulted in shedding of sporocysts by pups. The prepatent period varies in different studies from 12.0–15.3 days [44] to 9–10 days post infection (DPI) and the patent period varied from 18–85 DPI [43] to 9–11 DPI [45]. In the present study, infected esophageal muscles of cattle were fed to dogs, which began to shed sporocysts in the feces 7 days post-inoculation and repeated from 15–17 days post-inoculation. The sporocysts remained excreted but in low number until 30 days post-inoculation. The sporocysts then multiplied in quantity and continued to pass till the experiment ended (60 days post-inoculation). The findings are consistent with those of Park et al. [46], who showed that dogs were infected after being fed the heart muscle of slaughtered cattle in Korea. As shown in the present study, *Sarcocystis* sp. infect the kidney, liver, muscular esophageal tissue, and stomach of artificially infected dogs. These findings are nearly identical to those previously reported in *S. canis*-infected dogs with lesions that have been identified in the liver, lungs, skin, and brain, among other organs [47].

The histopathological findings in the current report are consistent with previous descriptions of fatal protozoal hepatitis associated with *S. canis*-like infection in multiple species of animals [48]. Our study showed that *S. cruzi* caused liver damage as indicated by total loss of normal hepatic architecture, necrotic tissue appearance, and inflammatory infiltrates, in accordance with others [49]. Earlier studies also indicated that the histopathologic hallmark of acute *sarcocystosis* is the presence of numerous *S. cruzi* schizonts in vascular endothelial cells of many organs, along with vasculitis, necrosis, and inflammatory infiltrates in several tissues including muscles, brain, and placenta [22]. Similar findings of central degeneration and necrosis have been described in *Sarcocystis idahoensis* infections of deer and mice [50].

The kidney is the second organ of choice in the current study. Light micrographs of kidneys of infected dogs indicated that *S. cruzi* induced histopathological changes and serious damage in the kidney cortex of the dog. These changes were apparent in both Malpighian corpuscle and renal tubules. Histopathological changes also include focal or diffuse mesangial proliferative glomerulonephritis, in accordance with the findings of others [51]. The occurrence of vasculitis and glomerulonephritis is suggested as a result of rupture of the cysts at irregular intervals with resultant release of antigens into circulation, thus forming antigen and antibody complexes that in turn get deposited in vessel walls or glomerular capillary basement membranes and cause damage [52]. Also, renal merogony and nephritis due to species of *Sarcocystis* have been reported in many intermediate hosts [53, 54].

Histopathological examination of our puppies' intestinal tissues showed edema, a marked increase in the number of goblet cells and destruction of intestinal villi. Similar results were reported by others [55, 56]. Earlier studies showed acute lesions such as edema, and necrosis development after ingestion of sporocysts and subsequent migration of sporozoites through body vessels [32]. In the present investigation, no inflammatory reaction was observed in the tissue. The absence of inflammatory response might be due to protozoa being housed in cysts inside muscle fibres, providing protection from host immunity, a theory that has been verified for a variety of parasites [32]. Our results are in line with the fact that inflammatory cells are not often reported in sarcocysts' infected tissues [35].

In the present study, sarcocysts isolated from the esophageal muscles of cattle in New Valley Governorate are genetically similar to the reported *S. cruzi* from other countries, which reflects in its prevalence in cattle in the New Valley region. A sequence comparison of the 18rRNA gene partial sequence of isolated New Valley sarcocysts showed identical nucleotide sequences to the reported *S. cruzi* from other countries, thus confirming its genetic relatedness and minimal genetic changeability [27, 57, 58]. Additionally, the RFLP analysis revealed similar electrophoretic and digestion separation patterns to New Valley isolated sarcocysts [59]. The molecular data reflect the viability and usefulness of the 18S rRNA gene as an important target for identification and characterization of *Sarcocystis* sp. from different hosts [60–62]. The phylogenetic pattern reveals high sequence similarity to other previously reported *S. cruzi* as indicated by their evolutionary distance patterns. To our knowledge, this is the first to report the prevalence of *S. cruzi* using TEM and 18S rRNA gene sequence analysis in native cattle from the New Valley Governorate, Egypt. Using morphological and molecular techniques such as light microscopy, TEM, PCR, and sequence analysis, the New Valley parasitic sarcocysts were identified as *S. cruzi.* Our findings also imply that the pathogenicity of livestock in the field should be given more consideration.

## 5. Conclusion

Results of our study contribute to updating the prevalence data concerning *S. cruzi* isolated from cattle in New Valley Governorate, Egypt. Morphological and molecular studies such as light microscopy, TEM, and 18S rRNA gene PCR amplification and DNA sequence analysis were used. The life cycle of the parasite and its histopathological changes inside the definitive host were examined. The present work verifies the genetic relatedness of New Valley Sarcocystis isolates to the previously reported *S. cruzi*. Investigations that highlight the presence of infections in humans are necessary to understand the risk of sarcocystosis, as well as for public health evaluations.

## Figures and Tables

**Table 1:** The pairwise genetic distance involving 19 *Sarcocystis* sp. including *Sarcocystis anasi* (EU55347) and *Sarcocystis falcatula* (MH626537) as an outgroup. Analyses were conducted using the maximum composite likelihood based on T92 + G + I model. The Egypt-NV obtained nucleotide sequence have been deposited in the GenBank with accession number: OL305830.

	Species	1	2	3	4	5	6	7	8	9	10	11	12	13	14	15	16	17	18	19
1	EU282016.1																			
2	AF176945.1	0.045																		
3	GQ251030.1	0.005	0.043																	
4	KC209743.1	0.033	0.041	0.032																
5	LC171828.1	0.057	0.047	0.055	0.045															
6	KJ917935.1	0.057	0.047	0.055	0.045	0.000														
7	KJ917906.1	0.057	0.047	0.055	0.045	0.000	0.000													
8	AB682779.1	0.057	0.047	0.055	0.045	0.000	0.000	0.000												
9	MN197848.1	0.057	0.047	0.055	0.045	0.000	0.000	0.000	0.000											
10	LC171829.1	0.057	0.047	0.055	0.045	0.000	0.000	0.000	0.000	0.000										
11	LC171827.1	0.057	0.047	0.055	0.045	0.000	0.000	0.000	0.000	0.000	0.000									
12	KP640133.1	0.057	0.047	0.055	0.045	0.000	0.000	0.000	0.000	0.000	0.000	0.000								
13	KC209738.1	0.057	0.047	0.055	0.045	0.000	0.000	0.000	0.000	0.000	0.000	0.000	0.000							
14	AB682780.1	0.057	0.047	0.055	0.045	0.000	0.000	0.000	0.000	0.000	0.000	0.000	0.000	0.000						
15	KT964025.1	0.059	0.049	0.057	0.047	0.002	0.002	0.002	0.002	0.002	0.002	0.002	0.002	0.002	0.002					
16	KT964021.1	0.061	0.051	0.059	0.049	0.004	0.004	0.004	0.004	0.004	0.004	0.004	0.004	0.004	0.004	0.005				
17	Egypt-NVE	0.057	0.047	0.055	0.045	0.000	0.000	0.000	0.000	0.000	0.000	0.000	0.000	0.000	0.000	0.002	0.004			
18	MH626537.1	0.062	0.072	0.062	0.070	0.064	0.064	0.064	0.064	0.064	0.064	0.064	0.064	0.064	0.064	0.066	0.068	0.064		
19	EU553477.2	0.060	0.074	0.060	0.072	0.066	0.066	0.066	0.066	0.066	0.066	0.066	0.066	0.066	0.066	0.068	0.070	0.066	0.005	

## Data Availability

This work is a part of the Ph.D. thesis submitted by Obaida F. Abo Elhussien to the New Valley University, Egypt, and certified in accordance with that work. Data used to support the findings of this study are available from the first and second authors upon reasonable request.
